# The Initial Factors with Strong Predictive Value in Relation to Six-Month Outcome among Patients Operated due to Extra-Axial Hematomas

**DOI:** 10.3390/diagnostics10030174

**Published:** 2020-03-23

**Authors:** Bartłomiej Kulesza, Jakub Litak, Cezary Grochowski, Adam Nogalski, Radosław Rola

**Affiliations:** 1 Department of Neurosurgery and Pediatric Neurosurgery, Medical University of Lublin, Jaczewskiego 8, 20-954 Lublin, Poland; kuleszabartek88@gmail.com (B.K.); rola.radoslaw@gmail.com (R.R.); 2 Department of Immunology, Medical University of Lublin, Chodźki 4a, 20-093 Lublin, Poland; 3 Department of Anatomy, Medical University of Lublin, Jaczewskiego 4, 20-090 Lublin, Poland; cezary.grochowski@o2.pl; 4 Laboratory of Virtual Man, Department of Anatomy, Medical University of Lublin, Jaczewskiego 4, 20-090 Lublin, Poland; 5 Department of Trauma Surgery and Emergency Medicine, Medical University of Lublin, Staszica 16, 20-090 Lublin, Poland; chirurgiaurazowalublin@tlen.pl

**Keywords:** traumatic brain injury, epidural hematoma, subdural hematoma, factors, outcome

## Abstract

Introduction: Traumatic brain injuries (TBI) are a real social problem, with an upward trend worldwide. The most frequent consequence of a traumatic brain injury is extra-axial hemorrhage, i.e., an acute subdural (SDH) and epidural hematoma (EDH). Most of the factors affecting the prognosis have been analyzed on a wide group of traumatic brain injuries. Nonetheless, there are few studies analyzing factors influencing the prognosis regarding patients undergoing surgery due to acute subdural and epidural hematoma. The aim of this study was to identify the factors which have the strongest prognostic value in relation to the 6-month outcome of the patients undergoing surgery for SDH and EDH. Patients and methods: The study included a group of 128 patients with isolated craniocerebral injuries. Twenty eight patients were operated upon due to EDH, and a group of 100 patients were operated upon due to SDH. The following factors from the groups were analyzed: demographic data, physiological factors, laboratory factors, computed tomography scan characteristics, and time between the trauma and the surgery. All of these factors were correlated in a multivariate analysis with the six-month outcome in the Glasgow outcome scale. Results: The factors with the strongest prognostic value are GCS score, respiration rate, saturation, glycaemia and systolic blood pressure. Conclusion: Initial GCS score, respiratory rate, saturation, glycaemia and systolic blood pressure were the factors with the strongest prognostic value.

## 1. Introduction

Traumatic brain injuries (TBI) are a real social problem, with an upward trend worldwide. TBI is the leading cause of death and disability, especially among young men. TBI is projected by the World Health Organization to become the third leading cause of global mortality and disability by the year 2020 [1]. The most frequent consequence of TBI is extra-axial hemorrhage, an acute subdural and epidural hematoma which usually requires a surgical treatment. Establishing a reliable prognosis after a head injury is difficult; as it is captured in the Hippocratic aphorism, ‘No head injury is too severe to despair of, nor too trivial to ignore’ [2]. The prognosis after this type of injury is difficult to predict, but usually unfavorable. Most of the factors affecting the prognosis have been analyzed on a wide group of traumatic brain injuries. There are only a few studies of the factors influencing the prognosis concerning patients undergoing the surgery due to acute post-traumatic subdural and epidural hematoma. The aim of this study was to identify the factors which have the strongest prognostic value in relation to the 6-month outcome of the patients undergoing surgery for acute post-traumatic subdural and epidural hematoma.

## 2. Materials and Methods

**Figure 1:**
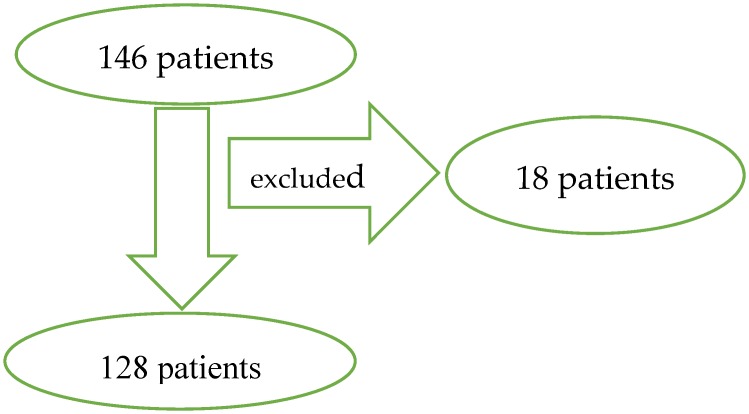
146 extra-axial hematoma patients required surgical treatment. Exclusion criteria included: the lack of complete medical documentation, incomplete laboratory tests, the lack of description of the computed tomography, lack of contact with the patient or his family after 6 months, and the occurrence of injuries other than head injury. At least 18 patient were excluded and 128 patient met inclusion criteria.

The study included a group of 128 patients with isolated craniocerebral injuries. The study obtained the consent of the Bioethics Commission located at Medical University in Lublin. Number of consent KE-0254/313/2016. Twenty eight patients were operated upon due to epidural hematoma (EDH) and 100 were operated upon due to acute subdural hematoma (SDH). All the patients were operated upon and treated in the Department of Neurosurgery from 1 October 2014 to 31 August 2017. During this period, 146 patients were treated surgically for extra-axial hematoma, whereas 18 patients were excluded from the study. Exclusion criteria for the examined group of patients included: the lack of complete medical documentation, incomplete laboratory tests, the lack of description of the computed tomography of the head, the lack of contact with the patient or his family after 6 months, and the occurrence of injuries other than head injury occurring in whole body computer tomography requiring treatment (Figure 1).

All of the factors were collected retrospectively after the admission of the patients into the Emergency Department. The following factors from the groups were analyzed: demographic data, physiological factors, laboratory factors, computed tomography scan characteristics and the time between trauma and surgery. The records were examined for demographic data, such as gender and age. Physiological factors included initial GCS score, pupil reaction to light, saturation, systolic blood pressure (SBP), heart rate (HR) and respiratory rate (RR). Laboratory factors included the number of white blood cells (WBC), hemoglobin (HGB) value, number of platelets (PLT), glycemia value, sodium concentration, coagulopathy and alcohol levels. Each patient included in the study had a computed tomography (CT) of their head as soon as it was possible. The study contained particular characteristics from the CT, such as the presence of skull fractures, subarachnoid hemorrhage (SAH), intraventricular hemorrhage (IVH), cerebral contusion, maximum thickness of the hematoma, midline shift (MLS) and the state of the basal cistern. The midline shift and maximum thickness of the hematoma were calculated using the OsiriX MD ver. 10.0 based on cross sections from the pre-operative CT scan. The last factor was the time between the injury and the surgery. 

All of the factors were correlated with a six-month outcome, according to the Glasgow Outcome Scale (GOS). After 6 months, a telephone call was made to the patient’s family member or the patient himself in order to obtain necessary information on the current state of health of the patient and, based on the conversation, the patient’s condition was determined according to the GOS scale. Subsequently, a favorable treatment result was assigned to the patients who presented 4 (moderate disability) or 5 (low disability) points on the GOS scale. The remaining patients with scores from 1 to 3 (death, vegetative state, severe disability), according to the GOS scale, received an unfavorable outcome. 

### Statistical Analysis

The results obtained from the tests were subjected to a statistical analysis. In univariate analysis, the Chi^2^ homogeneity test was performed to detect differences in six-month outcome between patients operated upon due to EDH or SDH. In the multivariate analysis, a logistic analysis was used to assess the factors with predominant prognostic value for the treatment outcome. A significance level of *p* < 0.05 was assumed to indicate the existence of statistically significant differences. In the logistic regression analysis, all the studied factors were considered. The dependent GOS variable was included in the dichotomic classification and assigned 1—unfavorable outcome (1 or 2 or 3 points on the GOS scale) and 0—favorable outcome (4 or 5 points on the GOS scale). The logistic analysis provided a significant model for the outcome. For the obtained model, the Chi2 value for the difference between the current model and the model with only the free expression was highly statistically significant (*p* < 0.0001). Additionally, Data Mining was used for the selection and elimination of variables to assess the prognostic factors of the outcome. The database and statistical research were based on the STATISTICA 13.0 computer software (StatSoft, Lublin, Poland).

## 3. Results

### 3.1. Group Presentation

The mean age of patients in the SDH group was 57.86 ± 18.26 years, and it was statistically significantly higher than the mean age of patients in EDH group—38.81 ± 13.37 years (*p* = 0.00001). In both groups, men were hospitalized most often (Table 1). The patients with SDH (median 6) had statistically significantly fewer points in the initial GCS scale than patients in ADH group (median 11.5) (*p* = 0.0006). In terms of physiological factors, abnormal values were more frequently observed in the SDH group than in the ADH group. Only in the case of saturation was this value statistically significant (*p* = 0,001). Laboratory factors did not differ significantly regarding the above-mentioned groups. In the case of one patient, hemoglobin > 18 mg/dL, sodium > 157 mEq/lt and glycaemia < 70 mg/dL were observed. It was demonstrated that skull fracture was more often associated with the patients in the EDH group (71.43%) rather than in the SDH group (30.00%), (*p* = 0.00007). It was shown that the basal cistern was more often compressed in the EDH group (53.57%) than in the SDH group (43.00%), (*p* = 0.03). The thickness of the hematoma was significantly greater in the EDH group (median 26.50 mm) than in the SDH group (median 17.00 mm), (*p* = 0.00004). Other factors from the computer tomography scan characteristics and the time between injury and the surgery did not differ significantly concerning the two groups.

### 3.2. Logistic Regression Analysis (Multivariate Analysis)

The factors that entered the model and were remarkable at the level of *p* < 0.05 were: the initial GCS scale, age, SBP and MLS. Those factors showed a significant relationship with the outcome. The obtained logistic model is presented below:

$$P\;\left(X\right)=\frac{e^{-4,357+0,62Initial\;GCS\;scale-0,087age+1,569SBP+3,248MLS}}{1+e^{-4,357+0,62OInitial\;GCS\;scale-0,087age+1,569SBP+3,248MLS}}$$

The positive factors estimated corresponding to the initial GCS score and SBP variables indicate that abnormal systolic blood pressure affects a poor outcome, while an increase in the GCS score improves it. However, the younger the patient, the more favorable the outcome. Using the unit odds ratio for the GCS variable (1.85), it was noticed that an increase of one point in the initial GCS score assessment improves the chances of obtaining a favorable outcome almost twice. A smaller midline shift than 10 mm increases the chances of obtaining a favorable outcome almost once (0.87). For the systolic blood pressure variable the odds ratio was 3.84, which means that the probability of an unfavorable outcome is almost four times higher for patients with systolic blood pressure lower than 89 mm Hg or higher than 141 mm Hg, compared with the patients presenting the normal pressure within 90–140 mm Hg. A one-year increase in the age of the patient increases the risk of a poorer outcome almost once (0.92) (Table 2). 

**Figure 2:**
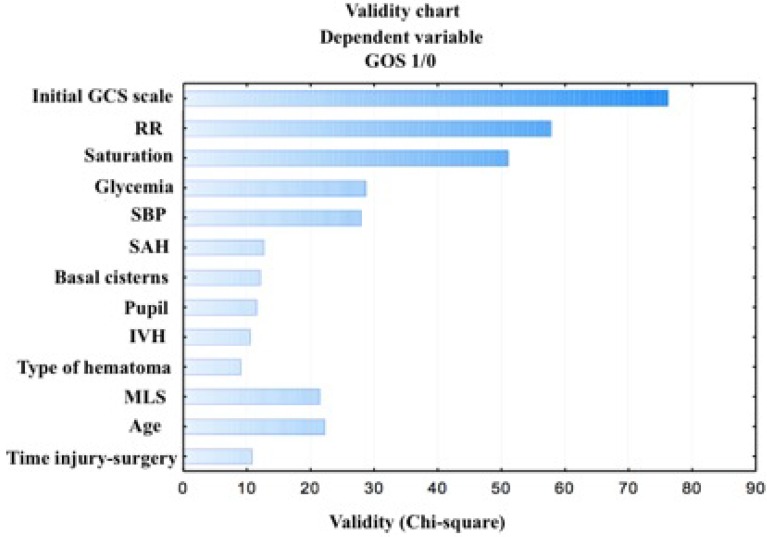
Dominant factors for the dependent variables of the outcome.

As a result of the analysis using Data Mining, the selection and the elimination of variables to assess the prognostic factors of the outcome, it was shown that the variables included in the table and the figures below are important variables for the assessment of the outcome in the GOS scale. The factors with the strongest prognostic value are: the initial GCS score, respiration rate under 12 or over 25 breaths per minute, oxygen saturation levels below or equal to 96 percent, hyperglycemia (>110 mg/dL) and systolic blood pressure beyond normal values (90–140 mm Hg). Other factors that were statistically significant (*p* ≤ 0.03) include: present SAH, compressed basal cisterns, reactive pupils, present IVH, type of hematoma (EDH or SDH), MLS greater than 10 mm, age, and time to surgery longer than 4 h (Table 3), (Figure 2).

### 3.3. Six-Month Outcome (Univariate Analysis)

**Figure 3:**
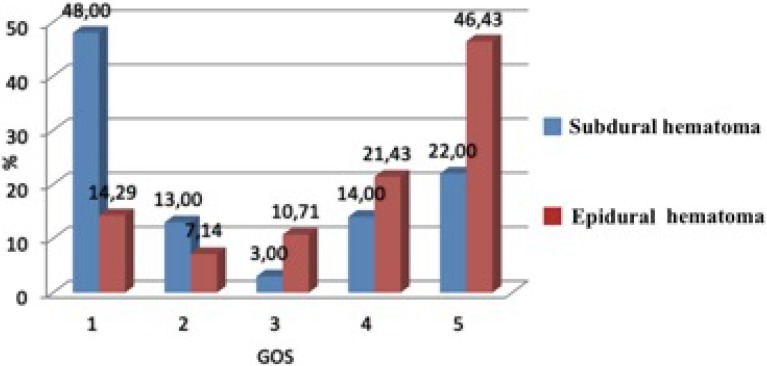
The percentage of patients in the groups according to their GOS score.

In the group operated upon for epidural hematomas, the patients were more likely to obtain a favorable outcome (67.86%) than those in the subdural hematoma group (36.00%). The differences found were statistically significant (Chi^2^ = 9.10, *p* = 0.003) (Figure 3).

## 4. Discussion

### 4.1. Demographic Data

Age is one of the strongest determinants of the outcome for TBI. Increasing age was associated with worse outcomes [1,3,4]. Authors suggested strong relation in groups after 60 y.o, some pointed at 40 y.o as a border age [5]. Age is the most powerful independent prognostic factor [4] and increasing age is associated with worse 6-month outcomes, creating an approximately linear function [6]. Similarly, the logistic regression analysis of our own study shows that an age increase of a year increases the risk of an unfavorable outcome almost once. The authors studying the patients with ADH and SDH also demonstrated that increasing age was associated with worse outcomes [7,8,9]. 

There is strong evidence that gender does not affect the prognosis in TBI [6,10,11]. In our study, gender was not placed among the factors which were significantly associated with the outcome after 6 months. This was similar in the case of authors who studied patients with EDH or SDH. [12,13,14]. 

### 4.2. Physiological Factors

The Glasgow Coma Scale (GCS) is used to assess the patient’s state of consciousness after a head injury, and it also has a strong prognostic value [1]. Age, followed by GCS motor score and pupil response, are the most powerful independent prognostic factors [4]. In addition, GCS, like age, creates a linear function [4,5]. The authors investigating the patients operated upon due to extra-axial hematoma also found that GCS score and pupil response had an important impact on the outcome [7,15,16]. The study confirmed that GCS on admission was the most powerful prognostic factor. Accordingly to analysis, GCS had the strongest prognostic value. One point increase on GCS was associated with almost two times better prognosis and favorable outcome. 

Dysautonomia is a condition in which the autonomic nervous system does not work properly. TBI causes dysautonomia, manifesting in episodes of fluctuations in blood pressure, heart rate, respiratory rate and others [1]. Hypotension and hypoxia following TBI are recognized as secondary insults associated with a poorer outcome [17]. Studies have shown that hypoxia causes rapid destruction of brain tissue and therefore, an unfavorable prognosis. Evidence that periodic optimization of oxygen supply for this category of patients is important [18,19]. Petroni et al. found a very strong relationship between hypotension and outcome. Hypotension (SBP < 90 mm Hg) was associated with unfavorable 6-month outcome in 96 percent of cases [18]. The results of randomized controlled studies showed that the increase in SBP is an important and independent factor of improved survival in patients with TBI presenting hypotensive. However, the increase in pressure should be maintained in people with normal blood pressure up to 140 mm Hg [19]. On the other hand, there is a characteristic U-shaped relationship between SBP and TBI outcome. The values of SBP higher than 135 (or even 150) mmHg, or lower than 90 mm Hg, were associated with poorer outcomes [4,10]. Recent guidelines, such as those from The Brain Injury Foundation, recommend maintaining systolic blood pressure above 90 mm Hg to optimize prognosis after TBI [20]. Kalayci et al. studied patients undergoing decompressive craniectomy for SDH. The authors of the study found that saturation less than or equal to 96% was significantly associated with unfavorable 6-month outcomes (*p* = 0.002) [20]. Both increase and the decrease of RR and HR beyond a normal range is associated with a poor outcome in TBI [10,21]. In our study, the analysis placed saturation, SBP and RR among factors with the strongest prognostic value regarding 6-month outcome. Additionally, abnormal SBPs will increase the chances of obtaining an unfavorable outcome by almost four times. In the case of HR, our study did not confirm a connection with the outcome. 

### 4.3. Laboratory Factors

Laboratory factors routinely recorded on admission following TBI had a predictive value. Hyperglycemia is a cause of secondary damage for patients after TBI, and it is associated with a poorer outcome [21]. Stress hyperglycemia is a common finding after the injury [22]. In our study hyperglycemia placed among the factors with the strongest prognostic value relation to outcome. Coagulopathy, especially prothrombin time and platelets, are major determinants of disability and death among patients with traumatic intracranial hemorrhage [21,23]. The presence of coagulopathy in ED was associated with the outcome [21,24]. Glucose and prothrombin time demonstrated a linear relationship with the outcome (increasing values associated with poorer outcome) [21]. Both hyponatremia and hypernatremia are associated with a poorer outcome, but hyponatremia is a relatively infrequent occurrence on admission following TBI. Sodium revealed a U-shaped relationship with outcome, but hyponatremia is more strongly related to a poorer outcome [1,21,25]. Hemoglobin and platelets levels showed an inverse linear relationship to the outcome (low values were associated with a poorer outcome) [21]. Leukocytosis was associated with a poor outcome after TBI [26]. Alcohol use was found to be an important risk factor for TBI, with the prevalence of alcohol intoxication being between 20–55% at the time of the injury [27,28]. Alcohol intoxication was associated with a poorer outcome after a severe TBI [28]. On the other hand, alcohol is associated with a lower mortality rate [29], precise mehanisms and relationship between alcohol use and the outcome after TBI required further investigation [27]. In our study, the statistical analysis did not show a significant relationship between coagulopathy, sodium level, hemoglobin concentration, WBC results, alcohol intoxication and outcome among patients with extra-axial hemorrhage.

### 4.4. Computer Tomography (CT) Scan Characteristics

Computerized tomography (CT) scanning provides an objective assessment of structural damage to the brain, and it is associated with the outcome following TBI [1]. Strong evidence was found for the midline shift [4,30,31], and a greater increase of the midline shift was associated with a poorer outcome [30]. The authors studying patients with EDH and SDH found that poorer outcomes were associated with a greater midline shift and the thickness of the hematoma [15,16,32]. Our analysis revealed that smaller MLS ratio correlates with better six month outcome. Skull fracture is not reliable in terms of predicting the outcome, but its presence is an obvious indication that the injury was caused by a greater force [33]. Skull fracture among TBI patients is associated with an increased risk of neurosurgically-relevant intracranial lesion [34] and unfavorable outcomes [35]. On the other hand, a certain amount of energy is absorbed when the skull is broken and consequently, the brain is not exposed to the full brunt of the impact [36]. The presence of a traumatic subarachnoid hemorrhage predicts poor outcome [13,30,35,37]. In our analysis using Data Mining, SAH and IVH are significantly associated with the outcome (*p* ≥ 0.001). This has also been noticed by other authors [9,13]. Obliteration of the basal cistern was associated with a poorer outcome after 6 months [4,5]. This has been also confirmed by our research in the analysis using Data Mining (*p* = 0.0005).

### 4.5. Time Injury-Operation

Matsushima et al. showed that in-hospital mortality was significantly lower in the group of patients operated upon up to 200 minutes after their arrival at the emergency department (*p* = 0,03) [38]. They came to similar conclusions [15,39]. Khaled et al. investigated patients with EDH, and stated that the time between a trauma and a surgery is the most important prognostic factor, and shortening this time to a minimum can reduce mortality to zero [11]. Seelig et al. based their study on patients undergoing surgery for SDH, and found that the surgery would reduce mortality from 90% to 30% within 4 h [40]. On the other hand, there are a few studies that did not associate a shorter period of time between the injury and the surgery with the outcome [13,41]. In the multivariate analysis, the time between the injury and the surgery was placed among the factors which were significantly associated with the outcome after 6 months (*p* = 0.03). Therefore, it is reasonable to perform a surgery as soon as possible [42,43,44,45]. 

## 5. Conclusions

Most of the studies were conducted on patients with a traumatic brain injury, and only a few of them studied a select group of patients operated upon due to extra-axial hematomas. We were unable to find a study which would collectively analyze all of the factors which we examined in one work regarding the patients operated upon due to epidural and subdural hematomas. It is interesting that the initial GCS, respiratory rate, saturation, glycaemia and systolic blood pressure were the factors with the strongest prognostic value. In addition to the GCS scale, these are factors that, with an appropriate treatment, could be normalized at the place of the accident, which may result in improved patient outcomes. These results require confirmation in other studies on a larger group of patients because this study has a significant limitation. The limitation of this study was the lack of inclusion of patients treated conservatively due to TBI. The number of patients was small, especially of those operated upon due to epidural hematomas and who had no other routine factors examined at admission, such as C-reactive protein and D-dimer. 

## Figures and Tables

**Table 1:** Demographic data.

		Groups			
Demographic Data		ADH (n = 28)			SDH (n = 100)
Sex		N	%	N	%
	Famale	2	7.14	14	14.00
	Male	26	92.86	86	86.00
Age	under 35 years	10	35.71	14	14.00
	36–60 years	17	60.71	42	42.00
	over 60 years	1	3.57	44	44.00
Age	mean ± st.dev.	38.82	±13.37	57.86	±18.26
	min–max	14–69		18–93	

st.dev.—standard deviation, min—minimum, max—maximum.

**Table 2:** Logistic model for the assessment of prognostic factors of the 6-month outcome.

	−95%CL	+95%CL	Chi^2^ Walda	Odd Ratio Unit	−95%CL	+95%CL	Rating
Constant	−8.0112	−0.7032	5.5722	0.0128	0.0003	0.4950	−4.3572
GCS	0.3417	0.8976	19.4777	1.8583	1.4074	2.4537	0.6197
Age	−0.1480	−0.0256	7.8846	0.9169	0.8625	0.9747	−0.0868
SBP	0.2635	2.4249	6.0629	3.8352	1.3015	11.3012	1.3442
MLS	−0.2558	−0.0324	6.5220	0.8658	0.7743	0.9681	−0.1441

Statystical analysis: Ch^2^ = 121.08; *p* < 0.0001.

**Table 3:** Dominant factors for the dependent variables of the outcome.

Factors	Chi^2^	*p*
Initial GCS scale	76,044	0.000000
RR	57,863	0.000000
Saturation	51,060	0.000000
Glycaemia	28,605	0.000000
SBP	27,953	0.000000
SAH	12,665	0.0004
State of basal cisterns	12,069	0.0005
Pupil reactive	11,509	0.0007
IVH	10,514	0.001
Type of hematoma	9059	0.003
MLS	21,398	0.003
Age	22,231	0.008
Time to surgery	10,783	0.03
